# Need for improving COVID-19 mortality registries: the case of Peru

**DOI:** 10.1186/s42506-021-00079-w

**Published:** 2021-06-16

**Authors:** Akram Hernández-Vásquez, Rodrigo Vargas-Fernández, Jesús Eduardo Gamboa-Unsihuay, Diego Azañedo

**Affiliations:** 1 Universidad San Ignacio de Loyola, Vicerrectorado de Investigación, Centro de Excelencia en Investigaciones Económicas y Sociales en Salud, Lima, Peru; 2 Universidad Científica del Sur, Lima, Peru; 3 Universidad Nacional Agraria La Molina, Lima, Peru; 4 Independent Researcher, Lima, Peru

To the Editor:

The SARS-CoV-2 pandemic has generated unprecedented health consequences with a record of nearly 117 million cases and more than two million deaths in 188 countries as of  July 2020 [1]. Peru was the 19th country in the world and fifth in Latin America [1] with the highest number of identified cases (1.5 million). In addition, there are 50 thousand deaths [2], and an excess of 355 deaths per 100,000 inhabitants by 2020 attributed to this condition compared to previous years [3]. Correct recording and monitoring of the dynamics of COVID-19 mortality are important to know the impact of the pandemic and the effectiveness of health measures, as well as to allow timely rethinking of these measures if necessary. However, under-registration of mortality has been identified in most Latin American countries, including Peru [4].

In Peru, the main sources of information on COVID-19 mortality are the National Informatics Deaths System (SINADEF in Spanish) and the Situational Room of the Ministry of Health of Peru (MINSA in Spanish). SINADEF is a virtual information system, in which medical personnel record the causes (basic, intermediate, direct, or intervening) of the death of patients, including those with suspected or confirmed COVID-19 cases [5]. In turn, the Situational Room of MINSA takes the National Center for Epidemiology, Prevention and Disease Control (CDC) as a source of information, which centralizes reports of all cases of death by COVID-19 verified by a laboratory test, recorded by health services at the national level through its systems of epidemiological surveillance and health intelligence [6].

According to the World Health Organization (WHO), for epidemiological surveillance purposes, death by COVID-19 is considered to occur as a result of a clinical picture compatible with COVID-19, whether a suspected or confirmed case [7]. Likewise, the Pan American Health Organization (PAHO) points out that the codes U07.1 (identified virus) and U07.2 (unidentified virus) of the International Classification of Diseases 10th edition (ICD-10) should be used to record the cases of deaths due to this disease [8]. Despite these recommendations, in July 2020, the Situation Room of MINSA reported the presence of an under-registration of more than 3000 deaths in the period from March to July 2020 compared to the SINADEF records [9]. This reflects a major problem that does not allow assessment of the actual impact of the pandemic according to the recommendations of the WHO and PAHO, generating confusion among the population and decision-makers.

To determine the extent of this under-registration throughout the national territory and to know where this problem is concentrated, an ecological study was carried out to estimate mortality by COVID-19 at the departmental level from codes U07.1 and U07.2 recorded in SINADEF (https://bit.ly/3hjRbOA) from the first reported case of COVID-19 in Peru to March 6, 2021, and compare the results with the figures reported by the Situational Room of MINSA in the same period (https://covid19.minsa.gob.pe/sala_situacional.asp). Furthermore, the code enabling the replicability of results in the R statistical program is available on the GitHub collaborative development platform (https://github.com/jeguns/ComparacionMortalidad).

A total of 94,574 COVID-19 deaths recorded under the ICD-10 codes were identified: U07.1 (n = 70,099) and U07.2 (n = 24,475) in SINADEF, while the Situational Room recorded 47,491 deaths by the same pathology (a difference of 47,088 deaths). This inconsistency in the number of deaths by COVID-19 recorded by SINADEF (using encoding U07.1 and U07.2) and the Situational Room of MINSA is also found in most departments that make up Peru. Lima is the department with the highest number of deaths reported by COVID-19 by both entities; thus, while MINSA reported a total of 20,770 deaths, SINADEF recorded 44,944, resulting in a difference of 24,184 deaths. Moreover, in the departments of Piura, the constitutional province of Callao, Ica, and Arequipa, there was a difference of more than two thounsands deaths when comparing the figures reported by MINSA and those estimated using the codes for COVID-19 of SINADEF. In addition, most departments of the mountains range have double or up to three times as many deaths according to SINADEF compared to MINSA. It should be noted that there are also 42 “no registration” department deaths in SINADEF (see Table 1).

**Table 1:** Comparison of the reported deaths from COVID-19 between the National Informatics Deaths System (SINADEF) and the Ministry of Health (MINSA) records (during the period from the first reported case of COVID-19 in Peru to March 6, 2021)

	SINADEF			MINSA
Departments	Confirmed COVID-19 deaths	Suspected COVID-19 deaths	Total COVID-19 deaths	Total COVID-19 deaths
Lima	32,273	12,671	44,944	20,770
Piura	3959	1725	5684	2429
Callao	3699	1378	5077	2515
Ica	3444	983	4427	2297
Arequipa	3025	1319	4344	2039
La Libertad	2649	841	3490	2879
Junin	2798	607	3405	1560
Lambayeque	2280	711	2991	2164
Ancash	2198	616	2814	1891
Cusco	1737	332	2069	767
Loreto	1005	846	1851	1212
Puno	1479	315	1794	638
Cajamarca	1571	215	1786	809
Huanuco	1254	248	1502	766
San Martin	1043	274	1317	899
Tacna	931	160	1091	611
Ucayali	677	331	1008	541
Ayacucho	759	127	886	516
Moquegua	579	256	835	461
Huancavelica	565	113	678	243
Tumbes	530	105	635	449
Apurimac	451	112	563	277
Pasco	407	70	477	249
Amazonas	360	75	435	329
Madre de Dios	400	29	429	180
Sin Registro	26	16	42	NA
Total	70,099	24,475	94,574	47,491

Data updated until March 06, 2021

*MINSA* Ministry of Health of Peru, *SINADEF* National Informatics Deaths System

These results show large differences between the figures reported by MINSA and SINADEF in all departments of Peru taking into account COVID-19 coding. These differences could be linked to the non-recording of deaths of suspected COVID-19 cases in a scenario of scarcity of diagnostic methods and operational capacity affecting various Latin American countries.

## Data Availability

The datasets analyzed during the current study are available in the SINADEF repository, https://bit.ly/3hjRbOA

## Abbreviations

SINADEF: National Informatics Deaths System
MINSA: Ministry of Health of Peru
WHO: World Health Organization
PAHO: Pan American Health Organization
COVID-19: coronavirus disease
